# Predictive Genetic Testing of Children for Adult-Onset Conditions: Negotiating Requests with Parents

**DOI:** 10.1007/s10897-016-0018-y

**Published:** 2016-09-28

**Authors:** Angela Fenwick, Mirjam Plantinga, Sandi Dheensa, Anneke Lucassen

**Affiliations:** 1 Clinical Ethics and Law, Faculty of Medicine, University of Southampton, Southampton, SO165YA UK; 2 Clinical Ethics and Law, Faculty of Medicine, University of Southampton, Room 4001, Building 85, Highfield Campus, University Road, Southampton, SO17 1BJ UK; 3 Wessex Clinical Genetic Service, University Hospital Southampton Foundation Trust, Southampton, SO16 6YD UK

**Keywords:** Genetic testing, Children, Clinical genetics, Professional perspectives, Decision-making

## Abstract

Healthcare professionals (HCPs) regularly face requests from parents for predictive genetic testing of children for adult-onset conditions. Little is known about how HCPs handle these test requests, given that guidelines recommend such testing is deferred to adulthood unless there is medical benefit to testing before that time. Our study explored the process of decision-making between HCPs and parents. Semi-structured interviews were conducted with 34 HCPs in 8 regional genetic services across the UK, and data were thematically analysed. We found that instead of saying ‘yes’ or ‘no’ to such requests, many HCPs framed the consultation as an opportunity to negotiate the optimal time of testing. This, they argued, facilitates parents’ considered decision-making, since parents’ eventual decisions after requesting a test was often to defer testing their child. In cases where parents’ requests remained a sustained wish, most HCPs said they would agree to test, concluding that not testing would not serve the child’s wider best interest. As a strategy for determining the child’s best interest and for facilitating shared decision-making, we recommend that HCPs re-frame requests for testing from parents as a discussion about the optimal time of testing for adult-onset disease.

## Introduction

Genetic testing can predict certain inherited conditions many years before their onset. When the onset is likely to be in adulthood, testing children before they are able to decide for themselves whether and when they wish to know about their inheritance can present healthcare professionals (HCPs) with ethical, social, and legal challenges. Professional guidelines have been issued to support HCPs in their decision-making. In Europe, these were first published in 1994 by the UK Clinical Genetics Society (CGS), (Clarke 1994) and in 1995 by the American Society of Human Genetics (ASHG) and American Society of Human Genetics (ACMG) (American Society of Human Genetics Board of Directors and American College of Medical Genetics Board of Directors 1995). These were followed by several others (Borry et al. 2009; British Society for Human Genetics 2010; Tozzo et al. 2012; Ross et al. 2013). The recommendations from these guidelines are well-established and have not changed significantly over time. Their primary message is that unless testing has current medical benefit, it should be deferred until a child is old enough to make her/his own decision (Fenwick 2010; Lucassen and Montgomery 2010; Anderson et al. 2015) protecting what Feinberg called the child’s right to an open future (Feinberg 1980; Bredenoord et al. 2014). Such guidance also aims to minimise potential psychological harm - to parents and the child - associated with knowing that the child is at risk of a serious condition, about which nothing can be done at present (Wade et al. 2013; Wertz et al. 1994).

Although professional consensus not to test in childhood for later-onset conditions has been consistent, there has been a noticeable change in emphasis as genetic technology has encompassed genomic testing. The ACMG’s (Ross et al. 2013) and the ASHG’s (Botkin et al. 2015) position statements both assert that predictive genetic testing may be ethically acceptable in certain circumstances, such as when it would resolve parental anxiety. According to Geelen et al. (2011), such guidance shifts attention away from a primary focus on protecting the child’s future autonomous choices towards promoting a discussion between professionals and parents about what is in the child’s ‘best interest’. This can be seen as a subtle move of focus from the future adult to the current child. The British Society for Human Genetics (BSHG) guidelines (2010) argue, for example, that professionals should aim at assessing, together with parents, the harms and benefits of testing and the ASHG position statement refers to ‘exploring reasons’ and to ‘thorough deliberation’ (Botkin et al. 2015). Despite this, there is nothing to suggest that testing would be recommended when there is no medical utility.

Experience from clinical practice and empirical studies suggests that, when a genetic condition is diagnosed in a family, many parents want their child tested to find out whether she/he is likely to develop a genetic condition in the future. There is evidence that parents request, or will want to request, such testing, despite the child having no symptoms of the condition in question and when there is no treatment or prevention available during childhood (Campbell and Ross 2005; Shkedi-Rafid et al. 2015). Indeed the advent of faster, cheaper genetic technologies and the perceived promise of more information from genomic testing likely means that requests from parents to test their children, before medical benefit, will increase in the future. Requests for predictive genetic testing for later-onset diseases is also increasingly encountered following other testing in a family. For example, incidental or additional findings from genomic testing in other family members may give rise to requests for testing in a child. HCPs must negotiate a difficult tension between an ethical obligation to encourage deferral of testing to enable the future adult to make their own choices and parents’ perceived rights to decide on behalf of their child, while ensuring they act in the best interest of the child (Roche and Berg 2015; Pelias 2006; Wilfond and Ross 2009).

Duncan et al. (2005) who surveyed clinical geneticists in the USA, Canada, UK, Australia, and New Zealand, found that clinicians sometimes provide testing for later-onset conditions for reasons beyond purely medical ones when confronted with parental requests. While we know this happens, little is known about how HCPs handle parental test requests in practice. How do they balance competing interests and for what reasons do they enable such testing in childhood? Understanding the difficulties they encounter, and how they weigh different factors to make a decision about whether testing would be in a child’s best interest, becomes increasingly important as genomic testing raises more challenges in this arena.

In this paper, we describe how HCPs responded to parents’ requests for predictive genetic testing in cases when there was no medical benefit at the time of testing. We explore how HCPs balanced different factors to come to a decision; how they communicated with parents about their requests; and how existing guidelines related to their decision-making practice. We examine approaches to a range of predictive genetic tests in children, including for reproductive risks, i.e. ‘carrier testing’.

## Methods

### Participants and Procedure

We sought the views of HCPs with experience in genetic testing and, in particular, those who had dealt with parents who had made requests for testing at a time when there was no medical benefit, and explored how they managed such requests in practice. HCPs were purposively sampled from UK professional groups (e.g., BSGM, CGS, and the UK Cancer Genetics Group), regional genetics services, and the UK Genethics Forum - a national group that meets regularly to discuss ethical issues arising from clinical practice. As the research progressed we adopted a snowballing strategy to improve recruitment. We sampled a mixed group because we did not intend to draw conclusions about any one profession, but rather about HCPs who face parental requests.

### Data Generation and Analysis

We used in-depth interviews to explore HCPs’ approaches to test requests and the extent to which these reflected current guidance. The interviews followed a semi-structured framework, which we developed with reference to existing literature and discussions with our patient and professional steering group. Interviews lasted 45–90 min, were digitally recorded and transcribed, and then data were thematically analysed to develop a conceptual framework of recurrent themes between and within participants’ accounts (Braun and Clarke 2013). We used an iterative process whereby emerging themes were explored in subsequent interviews to enable a better understanding of them and to explore differences and agreement between different HCPs. Quotes from participants are included to illustrate our findings and the professional background. Participant number (e.g. P1) is indicated for each quote. The research team operated as an interpretative community (Miller and Dingwall 1997) reviewing transcripts, conducting the analysis, and discussing emergent findings.

## Results

### Sample Characteristics

In total, our sample was 34 HCPs: 13 genetic counselors, 13 consultant clinical geneticists and 8 HCPs outside of clinical genetics but who were involved with genetic testing (e.g. pediatricians). Of these, 26 were female and 8 male, and 27 were based in England, 4 in Wales, and 3 in Scotland.

### Themes

We identified three key themes: (1) the future autonomy principle, (2) balancing no medical benefit and other factors in the assessment of best interests, and (3) reframing the request as when, not whether, to test.

- **Theme 1: The future autonomy principle**

Participants supported the key principle in guidelines (see, for example, BSHG 2010) that the protection of a child’s future autonomy was an important argument against testing (young) children for later-onset conditions. However, they varied in how strongly they held to the principle in their decision making in practice. There was general consensus that it should be applied when there was *clearly* no medical benefit at the time of the request for testing; for example testing a baby for Huntington disease or inherited breast cancer (e.g. BRCA1/2). Despite this general support, some HCPs thought that most young people/adults would not mind having had a predictive genetic test as a child without their input into the decision and, as such, the impact of a test result on a future adult’s autonomy could be overstated. However, as one HCP noted, for a few it would be an issue:

*The reason for not testing them is so they can make their own decision in the future. I would imagine that for 95% of them it’s not going to be a big issue as to whether or not they’re tested now as opposed to in the future and it’s us being more protective of them. But, of course, it’s that 5%, how big an issue is it going to be for them and I don’t think we know”* (Consultant, P21).

Given that we do not know who would mind at the time of the request for testing, adopting a general principle can be an appropriate and ethical approach.

HCPs differed in their application of the principle for (autosomal recessive) carrier testing. Some thought that a positive carrier test result was not the same as burdening a child with the knowledge of developing, or being at risk of, a medical condition in adulthood. Others argued that the principle should apply, on the basis that knowledge about carrier status is appropriate only at an age when potential carriers are making reproductive decisions or when they can understand the reproductive implications of the result. Predicting disease in a child’s future offspring was viewed as equally inappropriate and as lacking in medical benefit in childhood as predicting later-onset disease in the child:

*I don’t see that it’s any different from any other genetic test where it’s not going to have any [immediate benefit]. You can’t predict how somebody’s going to react to a result that somebody is a carrier or how they’ll understand it* (Counselor, P32).

The way HCPs interpreted guidelines played a key role in how they balanced harms and benefits of testing. Some HCPs were flexible in their interpretation, viewing guidelines as helpful *guidance.* Others adopted a very strict rule-based interpretation: unless there was clear identifiable medical benefit, the future autonomy principle was the default position. For example, one HCP argued that they had a firm policy of not testing *any* child under sixteen:

*We’ve had quite a number of requests for prediction work from minors, under 16, and it’s just a categorical no”* (Counselor, P26).

- **Theme 2: Balancing no medical benefit and other factors in the assessment of best interests**

All participants referred to an assessment of the child’s ‘best interest’ in decisions about testing but they also commented on how, in practice, they found it difficult to make such an assessment. They placed strong emphasis on medical criteria, taking into account the severity of the condition; how predictive the test was for the child’s future physical health; the difference between the child’s current age and the likely age of onset; and the availability of future treatment or interventions. In fact, many thought that medical criteria were the only criteria they themselves could reasonably apply to make a best interest judgement, identifying the non-medical factors that might contribute to the assessment of a child’s best interest to be largely outside their scope of knowledge:

*[The] reasons for testing other than for pure medical reasons are the ones that are very hard in practice, because they’re the reasons the medical profession has least insight into and the parent has most insight into… they’re the hardest to describe. So, if we’re saying in our guidelines that it is sometimes acceptable to do testing other than for medical benefit, if the parents can put forward a compelling argument for that testing to take place, then it’s quite hard for us to assess how compelling the argument is* (Consultant, P13).

HCPs therefore felt that parents’ role in any best interest assessment was an important one. For some this meant that test requests might be met because they believed that parents were acting in their child’s best interest:

*I think that parents on the whole do the best for their child that they can and if they feel that this is the way forward then that’s what I would support”* (Counselor, P17).

That is not to say that they felt that their role was simply to accede to parents’ wishes-- doing so might not ultimately be in the child’s best interest.

However, despite this perceived difficulty of identifying non-medical harms and benefits, in practice, most HCPs indicated that they did draw on factors beyond medical utility when considering best interests. HCPs found these factors compelling enough to over-ride the future autonomy principle and agree to test. These included the likely psychological and social impact of testing for the child; the parents’ experience of the genetic condition; whether parents seemed to understand that testing would reveal future (not current) risks; and the need to maintain a positive relationship with the child and family; for example:

*I think to have [a] blanket approach is quite damaging … we have to be more flexible with families and see that it’s not just about medical risk but actually it’s about the emotional side of things … in families and relationships together* (Counselor, P05).

In some cases, HCPs tested in childhood because they considered benefit to parents and the family would in turn benefit the child; for example the impact of parental anxiety on a child’s welfare in a best interest assessment. They were sympathetic to families with an extensive history of the condition for which they wanted their child tested; one HCP agreed to test the child of a parent whose deceased spouse and extended family had a history of heart disease:

*[I] just felt that she just had to know which of her children might be at the same risk as her husband with that … her anxiety levels were so high… so I ended up testing”* (Consultant, P13).

Similarly the anxiety of not knowing a child’s status could affect the parents’ relationship with them, so testing was considered to be the best interest of a child if it could improve this relationship for this HCP:

*…not knowing can affect bonding with young children and there’s been lots of parents that have reported that actually the anxiety makes it very difficult for them … Their parenting skills are affected this couple [who] … seriously needed to know and they weren’t sleeping…Testing someone to relieve an anxiety is not an unreasonable request* (Counselor, P19).

The potential to avoid parents treating a child differently, if they assumed that the child had a condition, was another example given by our participants: one tested a 9-year old child at 50 % risk of a faulty *P53* gene because:

*The family …couldn’t treat the child normally. Every time they had a headache, they took her to casualty. They were medicalising the child* (Counselor, P09).

Keeping the family engaged with the health service was deemed better for the child than the parents seeking private or commercial testing without medical support. This was also seen as a strong benefit to childhood testing:

*If we upset them [by saying ‘no’], we’re not helping the children in the long run either. That was another thing that persuaded me to test. An important thing was that the family were still engaged with us and would come back to talk about things* (Consultant, P34).

Finally, benefit to the family played out in different type of case where another child had previously been tested and the result was positive (e.g., carrier status). One participant articulated this in terms of testing on the grounds of equity:

*…when it comes to sharing information, the child who hasn’t been tested could feel left out…People all want to be offered the same and have the same opportunities* (Counselor, P19).

Interestingly, they did not weigh this ‘equity’ with the child having lost their ability to make decisions for themselves at a later date.

- **Theme 3: Reframing the request as when, not whether, to test**

As illustrated above, for many HCPs the decision-making process focussed on what they considered to be in the child’s best interest, taking into account medical and wider reasons. Another approach to requests for testing was to involve parents in discussion and negotiation about *when* genetic testing would be appropriate for their particular child or--to put it another way--when testing would be in their best interests. For example, in response to a question about how they would deal with requests to test children for conditions such as Huntington disease, one HCP stated:

*[the request would] be taken as an invitation to, let’s think about this disease and how you and your family are going to handle it…..so it’s just an opportunity to talk* (Consultant, P22).

HCPs expressed a number of connected reasons for re-framing the request as finding the best time for the test. First, it was seen as less adversarial: if parents came to clinic with a request, it would not help the patient-professional relationship for HCPs to immediately argue that testing was not permitted or possible. In such cases, HCPs reported that parents would often become more insistent and would not be willing to engage in further discussion or to consider the potential adverse consequences of testing:

*I would never say, ‘no’ that’s it, we’re not going to do it, because immediately you get their backs up and you’ve damaged the relationship before it starts* (Counselor, P05).

Another reason was that it created space for discussion about the pros and cons of testing which could include deferring testing to an age when the child would be old enough to be involved in the decision-making process:

*I usually try to establish quite early on that my job is not to sit here and refuse testing, it’s to look at all the aspects and arrive at a good decision that everybody is comfortable with, in the best interests* (Consultant, P16).

This helped to ensure the child’s ‘voice’ was not lost. Furthermore, it opened up discussion about the range of factors that could contribute to the best interest decision. Indeed, some HCPs saw their role as helping parents to make an informed decision about what was in the child’s best interest and understand why testing at that point might not be:

*They come along and say, “gene test, must-have…” But, once you make them aware that there are consequences… then they’re more aware of it. And I think we’ve had parents who have been very grateful for the extra conversation* (Consultant, P03).

Thus there was a recognition that when parents request testing they have not necessarily had the opportunity to talk through the pros and cons and the different options available to them. HCPs who engaged in discussions rather than agreeing or disagreeing with initial requests had often adopted this approach over time, commenting that when they had less experience they were more persuaded by parental rights or by the apparent rules that guidelines provided them. One argued that the greater the clinical experience a HCP had, the more comfortable and able they would be to engage in a meaningful discussion with parents and come to a mutual agreement:

*It’s very much people’s individual experience and how competent they feel in that situation. Some people enjoy exploring it with the family. Whereas others would much rather not. But that can work both ways, either completely withdraw and let the family do exactly what they want to do, or be paternalistic and say ‘no, you can’t have it because we’ve got guidelines’. You could avoid either way, having that discussion* (Counselor, P12).

Lack of experience might also make HCPs think they were legally required to provide the test if requested:

*Sometimes tests are done, I think, that may have more to do with the fear of being sued than [the test] being necessary* (Consultant, P32).

Ultimately, although HCPs reported that parents’ desire to test often changed after discussion, some parents remained insistent. When such wishes were sustained even after detailed discussions, most HCPs said they would grant the parents’ request, arguing that as long as such a request was informed, it was something the HCP should try to meet:

*At some point you feel that it’s their right to make that decision and you’re just there to help them make a decision, not to make the decision for them* (Consultant, P22).

In effect, sustained requests meant that HCPs might be more likely to give more weight to parents’ rights.

## Discussion

Our research provides an exploration of the way HCPs respond to parental requests for predictive genetic testing in children, and balance the different tensions that arise. We were interested in the process of decision-making between HCPs and parents and explored cases where parental desire for their children to be tested was particularly strong but where there was no immediate medical benefit to such testing. We focussed on how HCPs balanced different harms and benefits to come to a decision, how HCPs communicated with parents about their requests, and how this practice related to existing guidelines.

Our research showed that HCPs generally thought that a child’s future autonomy was an important factor when considering predictive genetic testing and recognised that it could be harmful to override it in childhood. Most HCPs acknowledged that the assessment of ‘best interests’ should encompass more than just medical benefit and, as such, might agree to testing. Empirical research and guidelines have also recognised the wider psychosocial benefits of testing in childhood; for example: the reduction of uncertainty and anxiety; the opportunity for psychological adjustment to the condition; the ability to make realistic life plans; and the sharing of information with family members (Botkin et al. 2015). However, it is notable that some of our participants reported feeling poorly equipped to judge anything other than medically-related harms and benefits and felt that parents were better able to make judgements about wider best interests. An assessment of best interest was challenging, as it was often difficult to separate out the best interests of an individual child and her/his family and identify who was best placed to decide. This finding adds weight to similar conclusions in two recently published studies which focussed on carrier testing (Noke et al. 2015; Vears et al. 2015). We concentrated more broadly on predictive testing showing that this is an issue for HCPs for a range of tests.

Re-framing parental requests in terms of finding the best timing for the test appeared a useful strategy for discussion around harms and benefits, including deferral of testing to when the child would be old enough to be involved in the decision-making process. In other words, it enabled HCPs and parents to engage in a complex discussion that involved both addressing an ethical principle and discussing the consequences of actions. By using this approach, HCPs tried to avoid the child’s ‘voice’ being lost or parents mixing their own interests with the child’s (Sarangi and Clarke 2002), while trying to facilitate parents’ considered decision making. As a result, in many cases, parents’ changed their first opinion--that testing should happen now--to an informed choice to defer testing. In some cases, parents’ requests remained sustained. HCPs were then more willing to grant the test because they were more confident that parents had evaluated the pros and cons of testing. This concurs with findings from previous research (Noke et al. 2015).

We agree with Geelen et al. (2011) that discussions should not be based on a narrow interpretation of best interests. However, for this to happen in practice, HCPs need to feel confident in their ability to make decisions. In a recent article, Birchley (2016) points out that discussions around what constitutes harm can lead to HCPs taking inconsistent approaches to deciding what is best for a child depending on the weight given to different factors at different times. He recommends articulating the values that underpin best interests. We would argue that this is also useful advice in the context of genetic testing: a consideration of the context of the request is important, but ethical principles such as safeguarding the future autonomy of the child should not be lost.

### Study Limitations

Our study is the first in recent years to investigate HCPs’ views about childhood testing. By adopting a qualitative approach and framing interviews using published guidelines, we have produced nuanced findings that have direct relevance to practice. One potential weakness is that HCPs were self-selecting and perhaps more likely than those who did not participate to have contemplated definitions of best interest and reasons for and against testing children. We did, however, purposely target recruitment of HCPs who had experience of parental requests for testing when there was no medical benefit. The majority of our participants were genetic HCPs, which could be seen as a limitation, since other specialties are increasingly involved in genetic and genomic testing, but we do not claim that our findings are generalizable. They may nevertheless have relevance to HCPs in other clinical settings.

### Conclusion and Research Recommendations

Our study focused on cases where parents requested a predictive test for a gene mutation already known about in their family. We recommend that HCPs adopt the strategy of finding the right time for testing, rather than focusing on whether or not to test. This more nuanced approach is more in line with the goal of genetic counseling: to facilitate informed decision-making. However, there is a challenge for the future when genome testing becomes part of routine clinical practice, as HCPs will face further pressures when parents request ‘additional findings’ indicating later-onset risks from an already sequenced exome or genome. One difficulty we predict is that this routinisation could likely mean that the time for considered decision-making is reduced at the same time as HCPs with less experience in managing such complex genetic issues offer tests. This is a challenge for future practice, which will be exacerbated if a condition is not already known about in the family and there is lack of time for pre-test information and counseling (Sukenik-Halevy et al. 2016). Further research is needed on the impact of these changes on ethical decision making around genomic testing in children, including decisions about when and how to disclose findings.

## References

[CR1] American Society of Human Genetics Board of Directors, & American College of Medical Genetics Board of Directors (1995). Points to consider: ethical, legal, and psychosocial implications of genetic testing in children and adolescents. *American Journal of Human Genetics, 57*(5), 1233–1241. PMC1801355 7485175

[CR2] Anderson JA, Hayeems RZ, Shuman C, Szego MJ, Monfared N, Bowdin S, Meyn MS (2015). Predictive genetic testing for adult-onset disorders in minors: a critical analysis of the arguments for and against the 2013 ACMG guidelines. Clinical Genetics.

[CR3] Birchley G (2016). The harm threshold and parents’ obligation to benefit their children. Journal of Medical Ethics.

[CR4] Borry, P., Evers-Kiebooms, G., Cornel, M. C., Clarke, A., Dierickx, K., & Public and Professional Policy Committee (PPPC) of the European Society of Human Genetics (ESHG) (2009). Genetic testing in asymptomatic minors: background considerations towards ESHG recommendations. *European Journal of Human Genetics, 17*(6), 711–719. doi:10.1038/ejhg.2009.25. 10.1038/ejhg.2009.25 PMC2947094 19277061

[CR5] Botkin, J. R., Belmont, J. W., Berg, J. S., Berkman, B. E., Bombard, Y., Holm, I. A., et al. (2015). Points to consider: ethical, legal, and psychosocial implications of genetic testing in children and adolescents. *American Journal of Human Genetics, 97*(1), 6–21. doi:10.1016/j.ajhg.2015.05.022. 10.1016/j.ajhg.2015.05.022 PMC4570999 26140447

[CR6] Braun V, Clarke V (2013). Successful qualitative research.

[CR7] Bredenoord, A. L., de Vries, M. C., & van Delden, H. (2014). The right to an open future concerning genetic information. *The American Journal of Bioethics, 14*(3), 21–23. doi:10.1080/15265161.2013.879952. 10.1080/15265161.2013.879952 24592834

[CR8] British Society for Human Genetics (2010). Report of a working party of the British Society for Human Genetics.

[CR9] Campbell E, Ross LF (2005). Parental attitudes and beliefs regarding the genetic testing of children. Community Genetics.

[CR10] Clarke A (1994). The genetic testing of children. Working party of the Clinical Genetics Society (UK. Journal of Medical Genetics.

[CR11] Duncan RE, Savulescu J, Gillam L, Williamson R, Delatycki MB (2005). An international survey of predictive genetic testing in children for adult onset conditions. Genetics in Medicine.

[CR12] Feinberg J, Aiken W, LaFollette H (1980). The child’s right to an open future. Whose child?.

[CR13] Fenwick A (2010). Are guidelines for genetic testing of children necessary?. Familial Cancer.

[CR14] Geelen, E., Van Hoyweghen, I., Doevendans, P. A., Marcelis, C. L., & Horstman, K. (2011). Constructing “best interests”: genetic testing of children in families with hypertrophic cardiomyopathy. *American Journal of Medical Genetics. Part A, 155A*(8), 1930–1938. doi:10.1002/ajmg.a.34107. 10.1002/ajmg.a.34107 21739592

[CR15] Lucassen, A., & Montgomery, J. (2010). Predictive genetic testing in children: where are we now? An overview and a UK perspective. *Familial Cancer, 9*(1), 3–7. doi:10.1007/s10689-009-9299-8. 10.1007/s10689-009-9299-8 19842063

[CR16] Miller G, Dingwall R (1997). Context and method in qualitative research.

[CR17] Noke M, Peters S, Wearden A, Ulph F (2015). A qualitative study to explore how professionals in the United Kingdom make decisions to test children for a sickle cell carrier status. European Journal of Human Genetics.

[CR18] Pelias, M. K. (2006). Genetic testing of children for adult-onset diseases: is testing in the child’s best interests? *The Mount Sinai Journal of Medicine, New York, 73*(3), 605–608. 16758098

[CR19] Roche MI, Berg JS (2015). Incidental findings with genomic testing: implications for genetic counselling practice. Current Genetic Medicine reports.

[CR20] Ross, L. F., Saal, H. M., David, K. L., Anderson, R. R., & American Academy of Pediatrics, & American College of Medical Genetics and Genomics (2013). Technical report: ethical and policy issues in genetic testing and screening of children. *Genetics in Medicine, 15*(3), 234–245. doi:10.1038/gim.2012.176. 10.1038/gim.2012.176 23429433

[CR21] Sarangi S, Clarke A (2002). Constructing an account by contrast in counselling for childhood genetic testing. Social Science & Medicine.

[CR22] Shkedi-Rafid, S., Fenwick, A., Dheensa, S., & Lucassen, A. M. (2015). Genetic testing of children for adult-onset conditions: opinions of the British adult population and implications for clinical practice. *European Journal of Human Genetics, 23*(10), 1281–1285. doi:10.1038/ejhg.2014.221. 10.1038/ejhg.2014.221 PMC4592073 25370041

[CR23] Sukenik-Halevy R, Ludman MD, Ben-Shachar S, Raas-Rothschild A (2016). The time-consuming demands of the practice of medical genetics in the era of advanced genomic testing. Genetics in Medicine.

[CR24] Tozzo P, Caenazzo L, Rodriguez D (2012). Genetic testing for minors: comparison between Italian and British guidelines. Genetics Research International.

[CR25] Vears DF, Delany C, Gillam L (2015). Carrier testing in children: exploration of genetic health professionals’ practices in Australia. Genetics in Medicine.

[CR26] Wade CH, Tarini BA, Wilfond BS (2013). Growing up in the genomic era: implications of whole-genome sequencing for children, families, and pediatric practice. Annual Review of Genomics and Human Genetics.

[CR27] Wertz DC, Fanos JH, Reilly PR (1994). Genetic testing for children and adolescents. Who decides?. JAMA.

[CR28] Wilfond, B., & Ross, L. F. (2009). From genetics to genomics: ethics, policy, and parental decision-making. *Journal of Pediatric Psychology, 34*(6), 639–647. doi:10.1093/jpepsy/jsn075. 10.1093/jpepsy/jsn075 18647793

